# Pathogenic Effects of IFIT2 and Interferon-β during Fatal Systemic Candida albicans Infection

**DOI:** 10.1128/mBio.00365-18

**Published:** 2018-04-17

**Authors:** Marcin Stawowczyk, Shamoon Naseem, Valeria Montoya, Darren P. Baker, James Konopka, Nancy C. Reich

**Affiliations:** a Department of Molecular Genetics and Microbiology, Stony Brook University, Stony Brook, New York, USA; b Biogen, Cambridge, Massachusetts, USA; Brown University

**Keywords:** ISG, chemokines, interferons, invasive candidiasis, reactive oxygen species

## Abstract

A balanced immune response to infection is essential to prevent the pathology and tissue damage that can occur from an unregulated or hyperactive host defense. Interferons (IFNs) are critical mediators of the innate defense to infection, and in this study we evaluated the contribution of a specific gene coding for IFIT2 induced by type I IFNs in a murine model of disseminated Candida albicans. Invasive candidiasis is a frequent challenge during immunosuppression or surgical medical interventions, and C. albicans is a common culprit that leads to high rates of mortality. When IFIT2 knockout mice were infected systemically with C. albicans, they were found to have improved survival and reduced fungal burden compared to wild-type mice. One of the mechanisms by which IFIT2 increases the pathological effects of invasive C. albicans appears to be suppression of NADPH oxidase activation. Loss of IFIT2 increases production of reactive oxygen species by leukocytes, and we demonstrate that IFIT2 is a binding partner of a critical regulatory subunit of NADPH oxidase, p67^phox^. Since the administration of IFN has been used therapeutically to combat viral infections, cancer, and multiple sclerosis, we evaluated administration of IFN-β to mice prior to C. albicans infection. IFN-β treatment promoted pathology and death from C. albicans infection. We provide evidence that IFIT2 increases the pathological effects of invasive C. albicans and that administration of IFN-β has deleterious effects during infection.

## IMPORTANCE

The attributable mortality associated with systemic C. albicans infections in health care settings is significant, with estimates greater than 40%. This life-threatening disease is common in patients with weakened immune systems, either due to disease or as a result of therapies. Type I interferons (IFN) are cytokines of the innate defense response that are used as immune modulators in the treatment of specific cancers, viral infections, and multiple sclerosis. In this study, we show using a murine model that the loss of a specific IFN-stimulated gene coding for IFIT2 improves survival following systemic C. albicans infection. This result infers a harmful effect of IFN during C. albicans infection and is supported by our finding that administration of IFN-β prior to invasive infection promotes fatal pathology. The findings contribute to our understanding of the innate immune response to C. albicans, and they suggest that IFN therapies present a risk factor for disseminated candidiasis.

## INTRODUCTION

During infection-induced sepsis, the protective effects of the innate immune response can turn deadly. If not kept in check, normal host defenses can promote morbidity. The opportunistic fungus Candida albicans is among the most common causes of infection in health care institutions, and disseminated candidiasis is linked to mortality rates of greater than 40% (1–6). Major risk factors include the use of antibiotics, catheters, chemotherapy, and surgical intervention. The efficacy of existing antifungal drugs is limited, and consequently there is an urgent need to explore the interplay between C. albicans and the host defense response to decipher mechanisms that enable or disable systemic fungal invasion (7–10).

Interferons (IFNs) are critical mediators of innate immune defense that were originally discovered for their protection against viral infections (11–14). Subsequently they were found to elicit various biological responses, including inhibition of cancer malignancies, and so their clinical use was expanded (11, 15). IFNs are also produced in defense response to bacterial and fungal infections; however, IFNs are reported to have both protective and deleterious effects during infection with these microbes. Type I IFNs (predominantly IFN-α and IFN-β) bind to the same cell surface receptor composed of two subunits, IFNAR1 and IFNAR2. Signal transduction by type I IFNs triggers the expression of hundreds of genes known as IFN-stimulated genes (ISGs) that confer varied biological effects in viral defense, differentiation, proliferation, and metabolism (16–19). In this report, we evaluated the role in invasive candidiasis of the IFN-induced gene with tetratricopeptide repeats 2 (IFIT2), also known as ISG54 (20). IFIT2 is a member of a family of ISGs with TPRs, structurally conserved motifs involved in multiple protein-protein interactions (21, 22). IFIT2 can form homodimers or stable complexes with two of its family members, IFIT1 and IFIT3 (23–25). Mice that lack the IFIT2 gene succumb to several negative-strand RNA virus infections, and expression of IFIT2 has been reported to inhibit proliferation and migration of cancer cells as well as promote their apoptosis (24, 26–30).

To determine the influence of IFIT2 during systemic candidiasis, we infected mice deficient in IFIT2 with C. albicans (31). The murine model of invasive candidiasis recapitulates the severe sepsis that can occur in clinical cases, resulting in a systemic hyperinflammatory response and renal failure (31). In contrast to the effect that IFIT2 loss has on host susceptibility to viral replication, we found that loss of IFIT2 improved host survival of invasive C. albicans infection. Comparative analyses of wild-type (WT) and IFIT2 knockout (*ifit2*^*−/−*^) mice revealed that *ifit2*^*−/−*^ mice respond with a heightened chemokine profile, a decrease in C. albicans titer, and improved overall survival. The loss of IFIT2 also correlated with increased reactive oxygen species (ROS) production in bone marrow cells, potentially contributing to survival of C. albicans infection. This result implied a negative effect of IFIT2 on ROS production, and mechanistic evaluation identified the cytosolic regulator of the phagocytic NADPH oxidase, p67^phox^, as a binding partner of IFIT2. These findings indicate induction of IFIT2 during the host response to systemic C. albicans infection contributes to increased disease severity.

Since IFIT2 is one of many proteins induced in response to infection and IFN, we tested the effects of IFN administration on pathogenesis driven by systemic candidiasis. Several previous studies investigated the role of type I IFN during systemic C. albicans infection by using mice that lack the IFNAR1 receptor gene. Differing results were reported: some studies indicated IFNAR1 signaling protected the host, whereas others found it promoted pathogenesis (32–37). We provide evidence in this study that administration of IFN-β has detrimental effects during invasive candidiasis by C. albicans.

## RESULTS

### IFIT2 knockout mice have improved survival of disseminated C. albicans infection.

**FIG 1:**
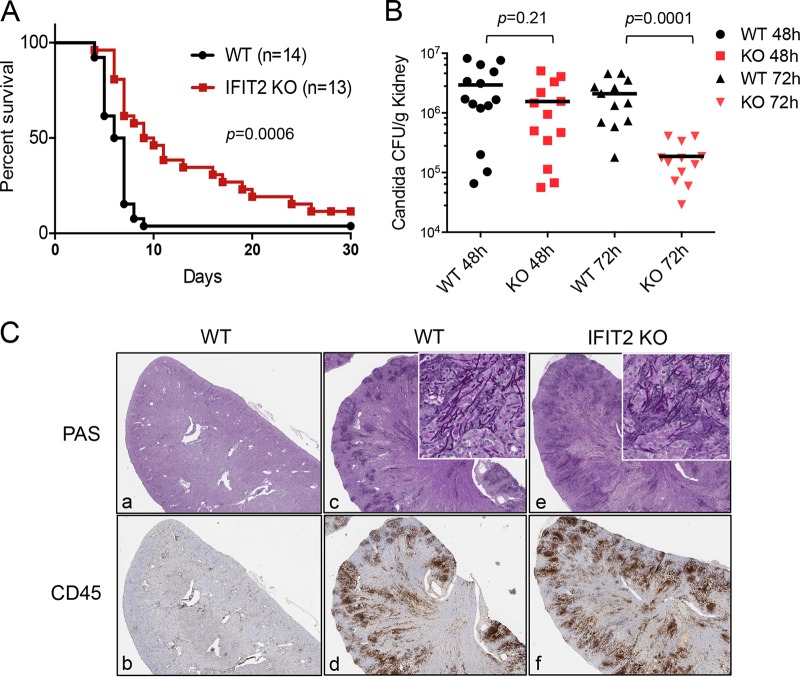
IFIT2 deficiency increases survival of C. albicans infection. (A) Wild-type (WT) or *ifit2*^*−/−*^ (IFIT2 KO) mice were infected intravenously with 2.5 × 10^5^ CFU of C. albicans. Kaplan-Meier survival curves demonstrate the significant increase in survival of IFIT2 KO animals. Four independent experiments were carried out with 3 to 4 WT and KO mice in each, and results were combined. *P* value determined by the log-rank test using with GraphPad Prism software. Four independent experiments were carried out. (B) C. albicans titers were measured in the kidneys of infected mice at 48 and 72 hpi and represent combined data from three independent experiments with 4 to 5 WT and KO mice. *P* values were determined using Student’s *t* test (mean ± standard error of the mean [SEM]). (C) Periodic acid-Schiff (PAS) stain for C. albicans (upper panel) and immunohistochemistry for infiltrating CD45 cells (lower panel) in kidneys from representative uninfected (a and b) and infected animals 72 hpi (c to f).

To determine the role of IFIT2 during C. albicans infection, we generated mice that lack expression of the IFIT2 gene with the Knockout Mouse Project (KOMP) Repository. The gene targeting scheme and expression of IFIT2 in *ifit2*^*+/+*^ (WT), *ifit2*^*+/−*^, or *ifit2*^*−/−*^ (knockout [KO]) mice are shown in Fig. S1 in the supplemental material. Previous studies with an independently generated IFIT2 KO strain reported the mice are susceptible to viruses with a negative-sense RNA genome (26). Likewise our IFIT2 KO strain displayed a decreased survival of infection with vesicular stomatitis virus (VSV) (Fig. S1). However, in contrast to their response to VSV, the IFIT2 KO mice had a different response to disseminated C. albicans relative to their WT counterparts (Fig. 1A). IFIT2 KO mice receiving a bloodstream C. albicans inoculum of 2.5 × 10^5^ CFU were better able to survive the infection. Ten days postinfection (p.i.), 95% of WT animals succumbed to infection, whereas nearly 50% of IFIT2 KO mice were alive. Thirty days postinfection, only 4% of WT mice were alive in contrast to 12% of the IFIT2 KO mice. Because renal insufficiency and kidney fungal burden correlate with lethality in this sepsis model, we evaluated kidneys of WT and IFIT2 KO mice (31, 38). Measurement of C. albicans titers in mice showed a decreased fungal burden in IFIT2 KO mice in comparison to WT mice at 24, 48, and 72 h postinfection (hpi) (Fig. 1B; see Fig. S2A in the supplemental material). Reduced kidney fungal burden in the IFIT2 KO mice appears to be predictive of survival. Western blot analyses demonstrated a clear induction of IFIT2 protein expression in WT kidneys during C. albicans infection (Fig. S2B). Although the average kidney weights in WT and IFIT2 KO mice were similar (Fig. S2C), fungal growth in IFIT2 KO mice was attenuated. The initial fungal burden was similar, but by 72 hpi, prior to signs of illness, the kidneys from IFIT2 KO mice had significantly reduced C. albicans CFU by 10-fold (Fig. 1B).

10.1128/mBio.00365-18.1 FIG S1  Murine IFIT2 gene knockout. Mice with a null allele in *ifit2*/*isg54* were generated by the NIH Knockout Mouse Project (KOMP) (Ifit2_AA5). (A) Diagram of *ifit2* deletion strategy and replacement of promoter and first exon with *lacZ-neo* cassette. KO generated in C57BL/6N mice from Charles River Laboratories, Inc. Fragment of chromosome 19 encompassing sequence 34,616,691 to 34,642,416 was deleted using insert consisting of *lacZ* gene and *loxP*-flanked *neo* cassette. The promoter, transcription start site, and first exon were deleted. Arrow indicates transcriptional start site. (B) Genotype results of eight mice either homozygous for wild-type (WT) *ifit2* alleles, or heterozygous or homozygous for knockout (KO) alleles. (C) IFIT2 mRNA expression measured in splenocytes by RT-PCR. (D) IFIT2 protein expression evaluated by Western blotting of murine splenocyte lysates prepared from homozygous WT *ifit2*^+/+^, heterozygous *ifit2*^+/−^, or homozygous KO *ifit2*^−/−^ mice (anti-mIFIT2 serum). Cells were untreated or treated with 1,000 U/ml murine IFN-β for 2 h. IFIT2 protein levels were similar in WT or heterozygous cells. (E) Kaplan-Meier survival curves of WT mice or IFIT2 KO mice following intranasal infection with vesicular stomatitis virus (VSV). Animals were sedated with isoflurane (Isothesia, Butler Schein) and infected intranasally with VSV in a 10-µl volume of complete Dulbecco’s modified Eagle’s medium (DMEM). VSV was obtained from ATCC, propagated in Vero cells, and the stock titer was determined by plaque assay. Survival of animals was monitored daily. Two independent experiments were performed, and a total of 12 WT and 12 IFIT2 KO mice were used. The graph shows results from one representative experiment with six WT and IFIT2 KO mice and 5 × 10^6^ PFU of VSV. The *P* value was determined by the log-rank test. Download FIG S1, PDF file, 0.9 MB. Copyright © 2018 Stawowczyk et al. 2018 Stawowczyk et al. This content is distributed under the terms of the Creative Commons Attribution 4.0 International license.

10.1128/mBio.00365-18.2 FIG S2  Response of WT and IFIT2 KO mice to C. albicans infection. (A) Kidney fungal burden in WT and IFIT2 KO mice at 24, 48, and 72 hpi. The means ± SEM of data from 4 independent experiments are shown at 24 hpi for 14 WT and 14 IFIT2KO mice, at 48 hpi for 24 WT and 22 IFIT2 KO mice, and at 72 hpi for 22 WT and 22 IFIT2 KO mice. (B) Western blot (WB) of IFIT2 protein expression in kidney lysates from uninfected (UN) or C. albicans-infected WT or IFIT2 KO mice. Kidneys were harvested 72 h postinfection, and polyclonal anti-mIFIT2 antibody was used to detect protein. Numbers represent individual mice. (B) Weights of kidneys isolated from WT (light bars) or IFIT2 KO (dark bars) mice uninfected or infected with C. albicans for 48 or 72 h. Download FIG S2, PDF file, 0.3 MB. Copyright © 2018 Stawowczyk et al. 2018 Stawowczyk et al. This content is distributed under the terms of the Creative Commons Attribution 4.0 International license.

C. albicans can grow as yeast or as invasive filamentous pseudohyphae and hyphae (39). To visually compare the forms of C. albicans in infected kidneys of WT and IFIT2 KO mice, histology was performed with periodic acid Schiff (PAS) staining (Fig. 1C). Although the fungal staining appeared modestly reduced in IFIT2 KO mouse kidneys 72 hpi, the filamentous forms were similar to those in WT kidneys. Immunohistochemistry with antibodies to CD45 (common leukocyte antigen) showed the inflammatory response to C. albicans and a modestly higher signal in the IFIT2 KO mice at this time postinfection.

### Increased proinflammatory chemokines in kidneys of infected IFIT2 KO mice.

**FIG 2:**
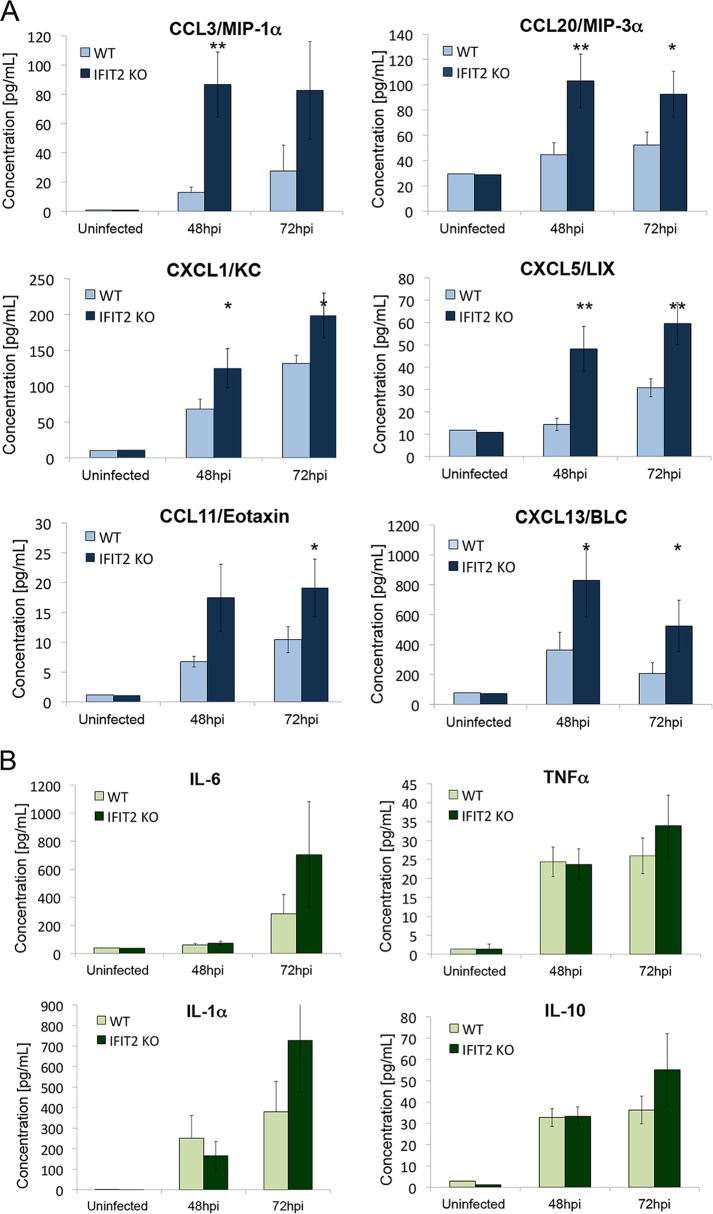
IFIT2 loss increases chemokine and cytokine levels in kidneys of C. albicans-infected mice. Mice were infected intravenously with 2.5 × 10^5^ CFU, at 48 and 72 hpi they were sacrificed, and kidney lysates were analyzed with LEGENDplex multianalyte flow assay kit. (A) Chemokine profiles from WT (light bars) and IFIT2 KO (dark bars) mice reveal higher levels of chemokines in infected IFIT2 KO mice. Table S1 lists the values of all chemokines measured. (B) Cytokine profiles from WT (light bars) and IFIT2 KO (dark bars). Table S2 lists values of all cytokines measured. Three to 4 independent experiments were performed with 3 to 6 mice in each, and data were combined. Significance of IFIT2 KO compared to WT using two-tailed Student’s *t* test (mean ± SEM). *, *P* < 0.05; **, *P* < 0.005.

The innate host defense to infection depends on the recruitment of inflammatory leukocytes; however, the cellular damage produced by a hyperinflammatory response can worsen fungal pathology (40–42). The inflammatory environment of infected kidneys was evaluated by measuring the levels of chemokines in uninfected mice or mice infected with C. albicans for 48 or 72 hpi. Kidney lysates were prepared and chemokines were measured by flow cytometry (BioLegend LEGENDPlex). Quantitation of a subset of chemokines is shown graphically in Fig. 2A, and the entire list of all chemokines tested and their corresponding values is presented in Table S1 in the supplemental material. Infected kidneys from the IFIT2 KO mice showed a significant increase in levels of a majority of the chemokines relative to WT mice early in infection. The functions of these chemokines include trafficking and activation of neutrophils, monocytes, macrophages, and lymphocytes (43). The improved survival of IFIT2 KO mice correlated with higher production of these regulatory chemokines.

10.1128/mBio.00365-18.6 TABLE S1  Chemokine profile of kidneys from uninfected (UN) and C. albicans-infected C57BL/6 WT mice and IFIT2 knockout mice 48 or 72 h postinfection. Mean values from 19 to 22 infected mice (5 independent experiments with 4 to 5 mice) are presented in picograms per milliliter as described in Materials and Methods. Download TABLE S1, PDF file, 0.1 MB. Copyright © 2018 Stawowczyk et al. 2018 Stawowczyk et al. This content is distributed under the terms of the Creative Commons Attribution 4.0 International license.

10.1128/mBio.00365-18.7 TABLE S2  Chemokine profile of kidneys from uninfected (UN) and C. albicans-infected C57BL/6 WT mice and IFIT2 knockout mice 48 or 72 h postinfection. Mean values from 19 to 22 infected mice (5 independent experiments with 4 to 5 mice) are presented in picograms per milliliter as described in Materials and Methods. Download TABLE S2, PDF file, 0.1 MB. Copyright © 2018 Stawowczyk et al. 2018 Stawowczyk et al. This content is distributed under the terms of the Creative Commons Attribution 4.0 International license.

The overall expression profile of cytokines in kidneys of infected mice was also measured. Quantitation of a subset of cytokines is shown graphically in Fig. 2B, and the list of all cytokines tested and their corresponding values is presented in Table S2 in the supplemental material. There was an elevation of some of the cytokines following C. albicans infection in both WT and IFIT2 KO mice. Several cytokines were elevated in IFIT2 KO mice compared to WT mice at 72 hpi, including interleukin-6 (IL-6), tumor necrosis factor alpha (TNF-α), IL-1α, and IL-10; however, due to varied levels between individual mice the differences did not reach statistical significance. There were no detectable differences in levels of IFN-β or IFN-γ between the WT and IFIT2 KO mice (Table S2).

**FIG 3:**
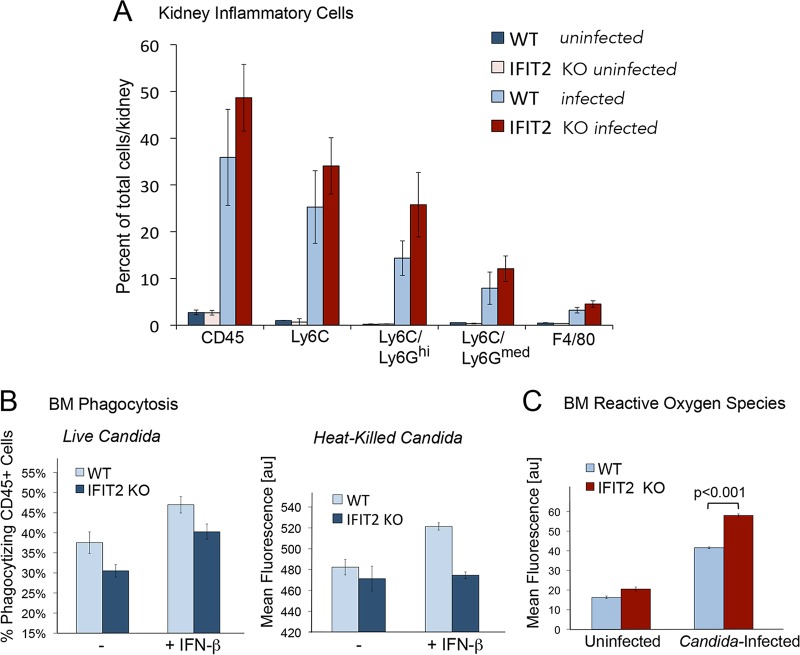
Kidney inflammation, bone marrow cell phagocytosis, and production of reactive oxygen species. (A) Profile of infiltrating inflammatory cells in kidneys from WT and IFIT2 KO mice. Cells were isolated from kidneys of mice left uninfected and mice infected with C. albicans for 72 h and analyzed by flow cytometry with antibodies to CD45 (all leukocytes), Ly6C (myeloid cells), Ly6G (neutrophils^high^ plus monocytes^medium^), or F4/80 (macrophages). The mean ± SEM of data was calculated individually from five infected WT or IFIT2 KO mice and two WT or KO mice left uninfected. (B) IFIT2 loss reduces phagocytosis of live C. albicans cells. (Left) Freshly isolated bone marrow (BM) cells from WT or IFIT2 KO mice were left untreated or were stimulated with IFN-β and incubated with live GFP-expressing C. albicans (*Ca*GFP) cells. The percentage of CD45-positive cells with *Ca*GFP fluorescence was measured by flow cytometry to evaluate efficiency of phagocytosis. Three independent experiments were performed with three mice. Results of a representative experiment are shown for the calculated mean ± SEM of data from 8 technical replicates. (Right) BM cells from WT mice or IFIT2 KO mice were left untreated or treated with IFN-β and incubated with heat-killed C. albicans cells stained with SYBR-Safe green. Propidium iodide was used to quench extracellular fluorescence, and mean fluorescence of phagocytized C. albicans was quantified by flow cytometry. One WT mouse and one IFIT2 KO mouse were used for each of 6 independent experiments. The means of data from the experiments were calculated, and statistical significance was determined by Student’s *t* test. (C) Loss of IFIT2 increases generation of reactive oxygen species. Bone marrow (BM) cells were isolated from WT or IFIT2 KO mice and were left untreated or infected with C. albicans *in vitro*. Cells were stained with DCFDA to measure reactive oxygen species. Four experiments were carried out with 10 technical replicates, and a representative experiment is shown. The *P* value was determined using Student’s *t* test (mean ± SEM).

Since IFIT2 KO mice displayed elevated levels of chemokines in infected kidneys, we measured the number of infiltrating leukocytes in WT or IFIT2 KO mice uninfected or infected with C. albicans (Fig. 3A). Flow cytometry was performed using antibodies to quantify CD45^+^ cells and the subset expressing Ly6C (neutrophils and monocytes), Ly6C/Ly6G^high^ (neutrophils), and Ly6C/Ly6G^medium^ and F4/80 (lymphocytes and macrophages). C. albicans infection increased the number of leukocytes in the kidneys of both WT and IFIT2 KO mice. Although there was a trend of increased infiltration of IFIT2 KO kidneys by inflammatory cells compared to WT kidneys, the values did not reach statistical significance.

### Bone marrow cells from IFIT2 KO mice have reduced ability to phagocytize C. albicans.

Phagocytes serve as a critical first line of defense against C. albicans (3, 44). Professional phagocytes express a number of pathogen recognition receptors (PRRs) that are stimulated in response to C. albicans, including Toll-like receptors (TLRs), C-type lectin receptors, and complement receptors (45, 46). Engagement of these receptors promotes engulfment of fungal cells and microbicidal action within phagolysosomes, as well as release of antimicrobial proteins. To determine the influence of IFIT2 on C. albicans phagocytosis, we measured the ability of freshly isolated bone marrow (BM) from WT or IFIT2 KO mice to phagocytize either live or heat-killed C. albicans. Phagocytosis of living C. albicans was measured by C. albicans green fluorescent protein (*Ca*GFP) expression (47). BM cells were labeled with antibodies to CD45 conjugated with allophycocyanin (APC) and incubated with GFP-expressing C. albicans in the presence or absence of IFN-β. Flow cytometry was used to calculate the percentage of CD45-positive cells with *Ca*GFP fluorescence. The percentage of CD45-positive cells with *Ca*GFP appeared significantly higher in BM samples from WT cells compared with IFIT2 KO cells, either with or without IFN-β treatment (Fig. 3B). Since this assay did not distinguish between *Ca*GFP-expressing cells engulfed and bound to the surface of cells, we evaluated phagocytosis of heat-killed C. albicans. Heat-killed C. albicans cells were stained with SYBR-Safe green fluorescent dye and incubated with BM cells left untreated or treated with murine IFN-β. Propidium iodide was added to quench extracellular green fluorescence, and flow cytometry was used to quantify green fluorescence per BM cell. Results indicate WT BM cells phagocytize heat-killed C. albicans more efficiently than IFIT2 KO cells following stimulation with IFN-β (Fig. 3B). Together the results indicate the loss of IFIT2 reduces phagocytosis of C. albicans.

### Loss of IFIT2 increases BM ROS production.

One of the mechanisms that phagocytes use to destroy C. albicans is the production of reactive oxygen species (ROS), known as the respiratory burst (44, 48–50). The primary enzyme complex responsible for ROS production is the membrane-associated NADPH oxidase that catalyzes the production of superoxide from oxygen and NADPH. Superoxide is converted into other damaging ROS, including hydrogen peroxide and hypochlorous acid. The importance of NADPH oxidase in fungal killing is evident in chronic granulomatous disease in which NADPH oxidase deficiency leads to susceptibility to recurrent fungal and bacterial infections (51). To evaluate the influence of IFIT2 on ROS production, we used the cell-permeable probe, 2′,7′-dichlorodihydrofluorescein diacetate (DCFDA) (52). Oxidation of DCFDA results in the formation of a fluorescent dichlorodihydrofluorescein (DCF) product that is trapped inside the cell and can be monitored using flow cytometry. BM cells were isolated from WT or IFIT2 KO mice, incubated with DCFDA, and remained uninfected or were infected with C. albicans. Both uninfected BM cells and infected BM cells from the IFIT2 KO mice produced elevated levels of DCF in comparison to WT mice, indicating increased ROS production (Fig. 3C). The increase in ROS could contribute to the reduced fungal burden and increased survival of IFIT2 KO mice following C. albicans infection.

### IFIT2 is a binding partner of p67^phox^.

**FIG 4:**
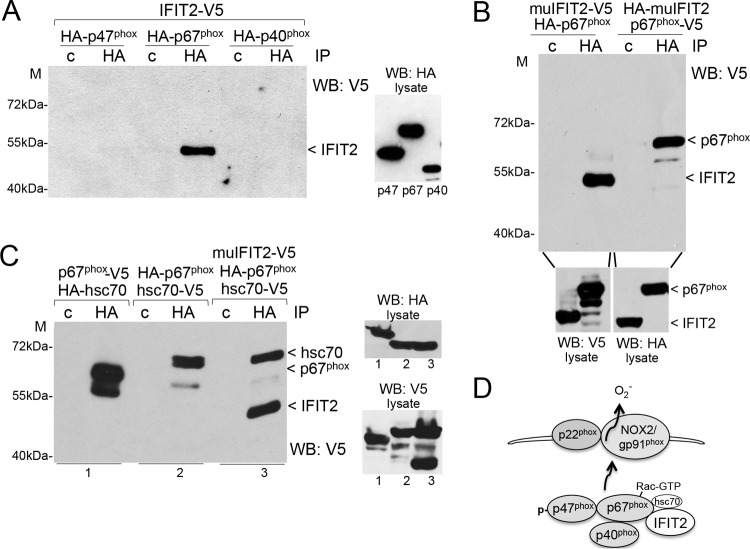
IFIT2 binding partners. (A) p67^phox^ is a binding partner of IFIT2. Association of IFIT2 with NADPH oxidase regulatory subunits was evaluated by cotransfection of 293T cells with DNA encoding V5-tagged human IFIT2 and HA-tagged human p47^phox^, p67^phox^, or p40^phox^. Cell lysates were prepared and immunoprecipitated (IP) with antibody to HA or a control (c) antibody. IFIT2 was detected in the immunocomplexes by Western blotting (WB) with V5 antibodies. Protein expression in cell lysates is shown in separate Western blot panel. M, molecular mass. (B) Murine IFIT2 and p67^phox^ are binding partners. Coimmunoprecipitation assays were performed as in panel A from cell lysates with HA- or V5-tagged murine IFIT2 coexpressed with V5- or HA-tagged murine p67^phox^. (C) Hsc70 binds p67^phox^ and IFIT2. Genes encoding human IFIT2, p67^phox^, and/or hsc70 were coexpressed with different epitope tags as indicated, and proteins were immunoprecipitated with HA antibody and detected by Western blotting with V5 antibody. (D) The diagram illustrates subunit regulation of NADPH oxidase, and identified binding of IFIT2 to p67^phox^.

The primary sources of cellular ROS are NADPH oxidases and mitochondria. NADPH oxidases are a family of enzyme complexes, and the phagocytic NADPH oxidase has been designated NOX2, or gp91^phox^ (49, 50, 53). NOX2 associates with p22^phox^ in plasma membranes, phagosomal membranes, and membranes of other intracellular vesicles. It is regulated by recruitment of cytosolic subunits p40^phox^, p47^phox^, and p67^phox^ and the Rac GTPase. Since the loss of IFIT2 resulted in increased ROS production, the inference was that IFIT2 may inhibit the activity of NOX2. To determine if IFIT2 interacts with the NOX subunits, we expressed hemagglutinin (HA) epitope-tagged human NOX subunits in cells with V5-tagged human IFIT2 and evaluated their association in immunocomplexes. Immunoprecipitation of HA-tagged NOX subunits identified specific association of IFIT2-V5 with p67^phox^ by Western blots (Fig. 4A). We also demonstrated physical association of the murine IFIT2 with murine p67^phox^ in immunocomplexes (Fig. 4B). Association of IFIT2 with p67^phox^ was confirmed using reciprocal epitope tags for coimmunoprecipitations. Since chaperone heat shock proteins have been reported to regulate NOX and to complex with signaling proteins on mitochondria in pathogen defense, we also tested the association of IFIT2 with members of the heat shock protein family (54–56). We found specific association of IFIT2 with hsc70, a constitutively expressed cytosolic chaperone (Fig. 4C; see Fig. S3A in the supplemental material). Moreover, we further identified hsc70 to be a binding partner of p67^phox^, suggesting that all three proteins may form a functional complex (Fig. 4C). Both p67^phox^ and hsc70 contain TPR domains, and it remains to be determined if specific binding to IFIT2 is mediated by TPR interactions. It is possible that the binding of IFIT2 to p67^phox^ and hsc70 regulates p67^phox^ recruitment to NOX2 or association with Rac (Fig. 4D).

10.1128/mBio.00365-18.3 FIG S3  hsc70 is a binding partner with IFIT2. (A) HeLa cells were transfected with HA-tagged hsp90, hsp70, or hsc70 expression plasmids and stimulated overnight with 1,000 U/ml human IFN-α. Endogenous IFIT2 was immunoprecipitated (IP) from cell lysates with rabbit anti-IFIT2 (24), and nonspecific rabbit antibody was used as a control (c). Heat shock proteins were detected in immunocomplexes by Western blotting with anti-HA antibodies. (B) Cells were transfected with V5-tagged hsc70 expression plasmid (lanes 1 and 2) or left untransfected (lanes 3 and 4) and stimulated overnight with 1,000 U/ml IFN-α. Antibody to IFIT2 was used to immunoprecipitate endogenous IFIT2 protein. The transfected or endogenous hsc70 was detected in immunocomplexes by Western blotting with anti-hsc70 antibody (Santa Cruz). (C) IFIT2 is a binding partner with Tom70. HeLa cells were left untransfected (lanes 1 and 2) or were transfected with V5-tagged Tom70 expression plasmid (lanes 3 and 4) and stimulated overnight with 1,000 U/ml IFN-α. Antibody to IFIT2 was used to immunoprecipitate endogenous IFIT2 protein. Both transfected Tom70 and endogenous Tom70 were detected in immunocomplexes by Western blotting with anti-Tom70 antibody (Santa Cruz). Download FIG S3, PDF file, 0.3 MB. Copyright © 2018 Stawowczyk et al. 2018 Stawowczyk et al. This content is distributed under the terms of the Creative Commons Attribution 4.0 International license.

### Monocyte/macrophage depletion identifies influence of IFIT2 on fungal burden.

**FIG 5:**
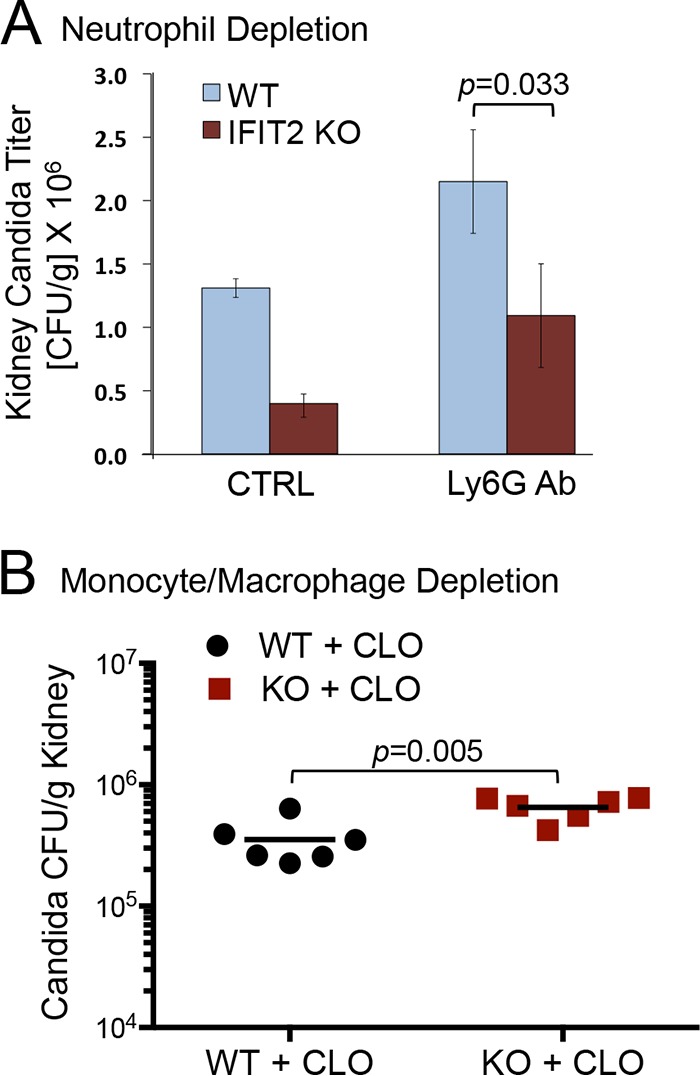
Monocyte/macrophage population of IFIT2 KO mice confers protective effects to C. albicans infection. (A) Depletion of neutrophils increases C. albicans titers, but the IFIT2 KO mice still have lower titers than WT mice. Antibody to Ly6G was administered intravenously to WT or IFIT2 KO mice 24 h prior to C. albicans infection, and kidney titers were compared with infected WT and IFIT2 KO mice that did not receive Ly6G antibody 48 hpi. Six antibody-treated and two untreated mice were evaluated individually for the WT or IFIT2 KO experimental group. *P* value was calculated by Student’s *t* test (mean ± SEM). (B) Depletion of monocytes/macrophages reverses the C. albicans titer difference in kidneys between WT and IFIT2 KO mice. WT or IFIT2 KO mice were treated intravenously with clodronate liposomes 24 h prior to C. albicans infection. Kidney titers were measured 24 hpi in each of six WT or KO mice as shown. Increase in kidney titer of IFIT2 KO mice compared to WT mice was significant using two-tailed Mann-Whitney test. **, *P* < 0.01.

Phagocytic cells are the first line of innate defense against C. albicans infection. To determine if the neutrophil or macrophage population is influenced by IFIT2, we tested depletion of these phagocytic subsets during infection of WT or IFIT2 KO mice. Anti-Ly6G antibody was administered to mice 24 h prior to infection with C. albicans to deplete the hematopoietic population of neutrophils. Neutrophil-depleted mice displayed accelerated behavioral signs of infection, and for this reason kidney fungal burden was measured at 48 hpi. The C. albicans titer was higher in both infected WT and IFIT2 KO animals treated with Ly6G antibody compared to mice lacking Ly6G treatment (Fig. 5A). A lower titer was still evident in IFIT2 KO mice compared to WT mice, indicating that although neutrophils were critical for defense against C. albicans infection, they were not responsible for the IFIT2 KO phenotype. The effectiveness of the Ly6G antibody was demonstrated by flow cytometry analysis of blood cells with Gr1 antibodies (see Fig. S4 in the supplemental material).

10.1128/mBio.00365-18.4 FIG S4  Evaluation of effectiveness of neutrophil depletion by anti-Ly6G administration. Anti-Ly6G antibodies were administered by intravenous injection 24 h prior to infection of WT (light bars) or IFIT2 KO (dark bars) mice with C. albicans. Mice were sacrificed 48 hpi, and the percentage of neutrophils in the blood was measured by flow cytometry with anti-Gr1 antibodies (BioLegend). Values are means ± SEM. Download FIG S4, PDF file, 0.1 MB. Copyright © 2018 Stawowczyk et al. 2018 Stawowczyk et al. This content is distributed under the terms of the Creative Commons Attribution 4.0 International license.

To evaluate the contribution of monocytes and macrophages to the reduction of fungal burden found in infected IFIT2 KO mice, we depleted these subsets in animals by intravenous administration of clodronate liposomes. WT and IFIT2 KO mice were treated with clodronate 24 h prior to infection with C. albicans. Clodronate liposomes kill monocytes and macrophages but do not affect neutrophils (57, 58). Both mouse strains showed accelerated behavioral changes indicative of infection as early as 24 h following infection, and for this reason, kidneys were harvested at that time to measure fungal burden. The depletion of monocytes and macrophages not only eliminated the difference in C. albicans titer between WT and IFIT2 KO mice, it resulted in a 2-fold increase of C. albicans CFU in WT mice compared to IFIT2 KO mice (Fig. 5B; Fig. S2A). These results indicate the loss of IFIT2 contributes to defense against C. albicans primarily by actions of monocytes and macrophages.

### IFN-β treatment promotes fatal pathogenesis of C. albicans infection.

**FIG 6:**
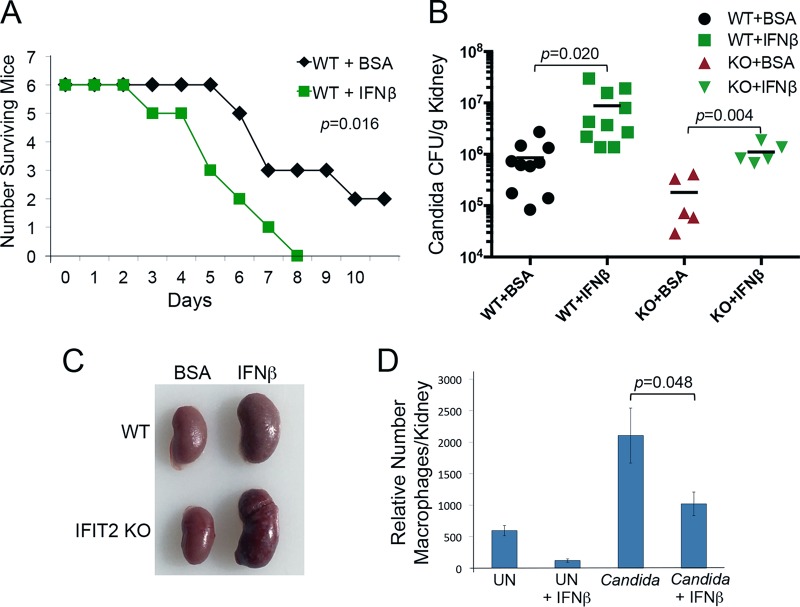
IFN-β treatment decreases survival of mice following C. albicans infection. (A) Kaplan-Meier survival curves demonstrate reduced survival of C. albicans infection following IFN-β treatment. WT mice were pretreated with 20 µg BSA or 1 × 10^5^ U murine IFN-β in BSA by intraperitoneal injection 24 h prior to C. albicans infection. Five mice were used per experimental group. *P* values were determined by the log-rank test. (B) IFN-β administration increases C. albicans titer. WT or IFIT2 KO mice were treated with IFN-β or BSA 24 h prior to C. albicans infection, and kidney titers were measured 48 hpi. *P* values were calculated by Student’s *t* test (mean ± SEM). (C) Appearance of kidney enlargement in WT or IFIT2 KO mice that were administered BSA or IFN-β prior to infection. (D) Treatment of mice with IFN reduces number of macrophages in kidneys. IFN-β (1 × 10^6^ U) was administered to mice, and 24 h later, they were infected with C. albicans or left uninfected (UN). Kidneys were harvested 48 hpi and analyzed by flow cytometry. Staining of macrophages was carried out with F4/80 antibodies. Numbers of macrophages were measured in a total of 10,000 kidney cells per sample (relative number of macrophages). Five infected mice and two uninfected mice were used per experimental group, and the mean is shown. *P* values were calculated by Student’s *t* test (mean ± SEM).

IFIT2 is one of hundreds of proteins produced in response to type I/III IFNs, and yet loss of this one gene had a significant effect on survival following C. albicans infection. The result suggests that type I IFNs could be detrimental to the fight against this pathogen. To test this hypothesis, we treated WT mice with IFN-β or control (bovine serum albumin [BSA]) 24 h prior to infection with C. albicans and measured survival. Animals treated with IFN-β had significantly increased mortality (Fig. 6A). All WT mice treated with IFN-β succumbed to C. albicans by 8 days p.i., whereas a third of BSA-treated mice were still alive at 11 days p.i.

To determine if the loss of IFIT2 still influenced pathogenesis in the context of IFN-β treatment and induction of all other ISGs, we compared kidney fungal burdens in WT and IFIT2 KO mice pretreated with IFN-β. Mice were administered BSA or IFN-β 24 h before infection, and C. albicans growth was measured 24 hpi. IFN-β treatment increased C. albicans replication in both WT and IFIT2 KO mice, but the relatively lower titer in IFIT2 KO mice remained evident (Fig. 6B). These data indicate that the contribution of IFIT2 has a significant effect on C. albicans pathogenesis during an IFN response.

Visual inspection of infected kidneys from WT or IFIT2 KO mice treated with IFN-β revealed a dramatic change (Fig. 6C). Kidneys from both WT and IFIT2 KO mice treated with IFN-β were swollen compared to those from BSA-treated controls, correlating with the increased fungal burden. The average kidney weights of IFN-β-treated mice were also increased (data not shown). To evaluate leukocyte infiltration of uninfected or C. albicans-infected kidneys from WT mice untreated or pretreated with IFN-β, we isolated cells for analysis by flow cytometry. Fluorescent antibodies to CD45 and lineage-specific markers indicated there were no significant changes in the total number of infiltrating leukocytes (data not shown). However, flow cytometry analysis with antibody to F4/80, which is expressed at high levels on various macrophages, did show that IFN-β treatment significantly lowered the relative number of macrophages in the kidneys of both uninfected and infected mice (Fig. 6D). IFN-β administration lowered the macrophage population by more than 50%. The result suggests that IFN-β may contribute to the pathogenic effects of C. albicans infection by reduction of macrophage survival, proliferation, or recruitment to the kidneys.

**FIG 7:**
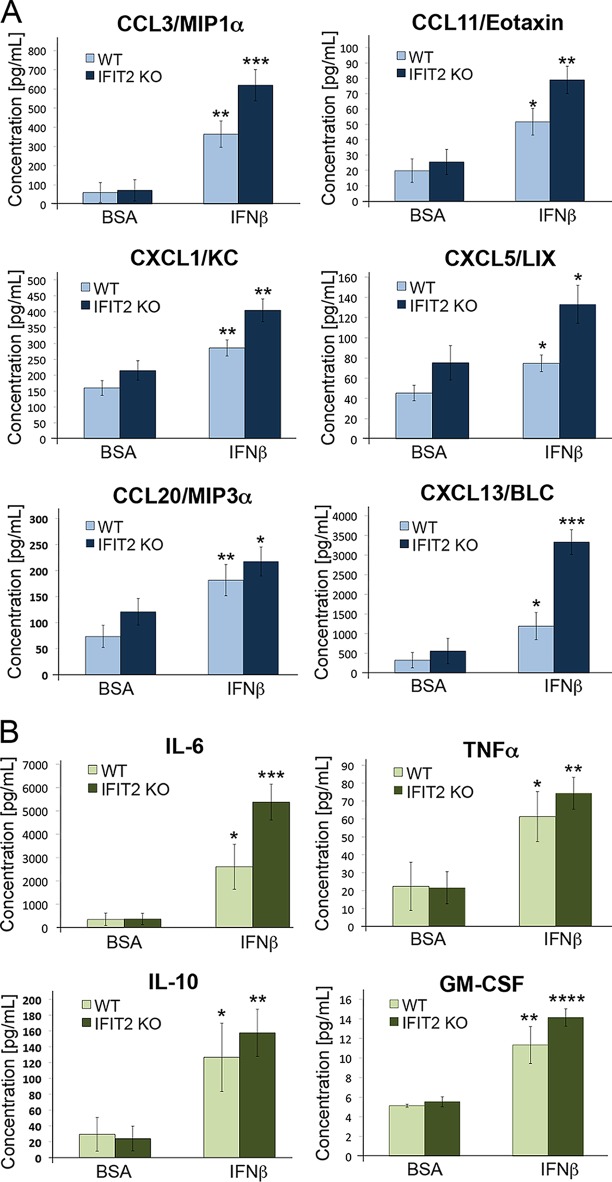
IFN-β effect on kidney proinflammatory chemokines and cytokines. Kidney lysates were prepared and analyzed using the LEGENDplex multianalyte flow assay kit. Five mice were individually evaluated individually per experimental group. (A) Chemokine profile of kidneys from WT (light bars) and IFIT2 KO (dark bars) mice. Mice were administered BSA or murine IFN-β 24 h prior to C. albicans infection, and kidneys were harvested 48 hpi. Table S3 lists values of all chemokines measured. (B) Cytokine profile of kidney lysates described in panel A from WT (light bars) and IFIT2 KO (dark bars) mice pretreated with BSA or IFN-β prior to infection. Table S4 lists values of all cytokines measured. Significance is shown for IFN-β treatment compared to control BSA-treated mice using the two-tailed Student’s *t* test (mean ± SEM). *, *P* < 0.05; **, *P* < 0.01; ***, *P* < 0.001; ****, *P* < 0.0001.

IFNs have been shown to drive the production of various chemokines and cytokines that influence inflammatory and adaptive immune responses (59–61). To establish the effect of IFN-β administration on the profile of chemokines and cytokines in kidneys of C. albicans-infected mice, we analyzed kidney lysates using a flow cytometry-based assay (BioLegend LEGENDPlex). IFN-β treatment of infected mice led to a significant increase of many chemokines in comparison to BSA-treated controls (Fig. 7A; see Table S3 in the supplemental material). Strikingly, even with IFN-β treatment, the infected IFIT2 KO mice showed higher concentrations of chemokines than the infected WT mice. The trend was similar for the cytokine profile (Fig. 7B); however, IL-6 was the only cytokine with a statistically significant increase in infected IFIT2 KO mice versus infected WT mice following IFN-β treatment (see Table S4 in the supplemental material). Together these results indicate that administration of IFN-β promotes an increase in inflammation coincident with decreased survival and increased fungal burden.

10.1128/mBio.00365-18.8 TABLE S3  Chemokine profile of kidneys from C57BL/6 WT mice and IFIT2 KO mice treated with BSA or IFN-β and subsequently infected with C. albicans for 72 h. Mean values from 5 infected mice are presented in picograms per milliliter as described in Materials and Methods. Download TABLE S3, PDF file, 0.1 MB. Copyright © 2018 Stawowczyk et al. 2018 Stawowczyk et al. This content is distributed under the terms of the Creative Commons Attribution 4.0 International license.

10.1128/mBio.00365-18.9 TABLE S4  Cytokine profile of kidneys from BSA- or interferon-β (IFN)-treated C57BL/6 WT mice or IFIT2 KO mice infected with C. albicans for 72 h. Mean values from 5 infected mice are presented in picograms per milliliter as described in Materials and Methods. Download TABLE S4, PDF file, 0.1 MB. Copyright © 2018 Stawowczyk et al. 2018 Stawowczyk et al. This content is distributed under the terms of the Creative Commons Attribution 4.0 International license.

## DISCUSSION

An effective innate immune response is essential to successfully defend against fungal infections. In this study, we investigated the role of a type I IFN-induced protein, IFIT2, during systemic C. albicans infection. Unexpectedly, IFIT2 KO mice displayed a significantly higher survival rate than WT mice, and this correlated with a decrease in kidney fungal burden (Fig. 1). This response indicates IFIT2 contributes to the fatal pathology of C. albicans systemic infection.

The inflammatory response to C. albicans infection is critical for successful fungal clearance; however, virulent strains are associated with a hyperinflammatory response (42). Studies to investigate the role of specific cytokines and chemokines in the response to infection have shown that these mediators can be beneficial (e.g., IL-6, TNF-α, IL-1, and CXCR1) (62–65), or they can be detrimental (CCR1) (66). IFNs are known for their protective effects during infectious disease, yet it is becoming more apparent that IFNs also can have detrimental consequences that exacerbate pathology (13, 59, 60). IFNs can promote production of chemokines and cytokines in a hyperinflammatory response that leads to tissue damage. To assess immune cell activation and recruitment, we measured chemokine and cytokine profiles during infection. The IFIT2 KO mice, in comparison to WT mice, produced significantly higher levels of proinflammatory chemokines such as CCL3, CXCL1, and CXCL5 during systemic infection with C. albicans (67) (Fig. 2A; Table S1). The increased synthesis of chemokines in the IFIT2 KO mice during systemic infection may promote the recruitment or activation of phagocytes and thereby contribute to survival and reduced fungal burden. Immunohistochemistry and flow cytometry analyses showed a trend of higher leukocyte infiltration of IFIT2 KO mouse kidneys.

The profiles of cytokines in WT compared to IFIT2 KO mice were only modestly different, although IFIT2 KO mice showed elevated induction of proinflammatory mediators, such as IL-1α and IL-6 (Fig. 2B; Table S2). The levels of IFN-β were similar in the WT and IFIT2 KO mice, indicating IFIT2 does not have a significant influence on the production of IFN-β. Notably, the levels of IL-17, a cytokine that has been shown to be essential for protective immunity during chronic mucocutaneous candidiasis (68), were similar in IFIT2 KO and WT mice and actually lower than those in uninfected mice.

Since the production and action of reactive oxygen species (ROS) are essential for antimicrobial defense of phagocytes, we measured ROS produced by BM leukocytes from WT and IFIT2 KO mice. C. albicans increased ROS production in cells of both WT and IFIT2 KO mice; however, cells isolated from IFIT2 KO mice displayed statistically higher levels of ROS, both prior to and after infection (Fig. 3C). Production of ROS may not be the only mechanism by which IFIT2 KO mice reduce fungal loads, but the higher levels of ROS do correlate with IFIT2 KO survival and lower kidney fungal titer. The increased ROS was evident in spite of the fact that C. albicans expresses catalase and superoxide dismutases that can block the toxic effects of cellular ROS (69).

NADPH oxidases and mitochondria are two primary sources of cellular ROS, and are responsible for antimicrobial defense and signaling (44, 48–50, 56, 70, 71). The NADPH oxidase that is prevalent in phagocytic cells is designated gp91^phox^, or NOX2, and is associated in plasma and phagocyte membranes with p22^phox^ (50, 53). Activation of NOX2 occurs following the translocation and association with a cytoplasmic complex of phosphorylated p47^phox^, p67^phox^, p40^phox^, and Rac-GTP. Since the C. albicans-infected BM cells from the IFIT2 KO mice elicited higher ROS levels than WT cells, we postulated a potential IFIT2 mechanism of action that includes binding of IFIT2 with one of the subunits of the NADPH oxidase. This was demonstrated by specific coimmunoprecipitation of IFIT2 with p67^phox^, a key regulatory protein that binds Rac-GTP and NOX2 (Fig. 4) (72). This finding suggests the possibility that IFIT2 binds to p67^phox^ and inhibits its ability to stimulate or associate with other subunits of the oxidase. Since NOX enzyme activity is regulated by heat shock proteins (54, 55), we evaluated the association of heat shock proteins with IFIT2. We found IFIT2 to be a binding partner of hsc70, a constitutively expressed heat shock protein 70 (hsp70) family member with TPR motifs (73). In addition, we demonstrated hsc70 binding to p67^phox^, suggesting that IFIT2, hsc70, and p67^phox^ may cooperate to modulate NOX enzyme activity (54, 55).

Heat shock proteins not only influence NOX activity, but they impact the redox state of the cell by controlling classical mitochondrial import. Since hsc70 is known to bind TOM70, a component of the mitochondrial import machinery (74, 75), and TOM70 contains TPR motifs, we tested the interaction of IFIT2 and TOM70. Specific coimmunoprecipitation indicated IFIT2 also associates with TOM70 (Fig. S3C). Therefore, it is possible that IFIT2 influences ROS production by both modulation of NADPH oxidase and mitochondrial protein import, especially since the TPR motifs of IFIT2, hsc70, and TOM70 could influence their association. Invasive C. albicans infections are prevalent in patients with chronic granulomatous disease caused by mutations in phagocyte NADPH oxidase subunits and the consequent reduced ROS. The improved survival and reduced fungal burden in IFIT2 KO mice systemically infected with C. albicans may reflect protective effects of enhanced ROS production.

To determine if leukocytes from the BM of IFIT2 KO or WT mice showed a differential antifungal activity *in vitro*, we incubated freshly isolated BM cells with C. albicans overnight and quantified colony growth. Antifungal activity of BM cells *in vitro* from WT or IFIT2 KO mice was measured; however, no statistically significant difference was detected (see Fig. S5 in the supplemental material). In addition, although IFN-β treatment of infected mice increased pathology, *in vitro*, IFN-β promoted antifungal activity of BM cells. These results indicate that the complexity of immune defense *in vivo* during invasive C. albicans infection cannot be reproduced *in vitro* under the conditions described. The ROS produced *in vivo* may serve to destroy pathogens, but may also serve as critical signaling molecules in the activation or recruitment of cells and mediators of defense. Differential chemokine/cytokine profiles affect responses of various recruited inflammatory cells and kidney cells in the animal that are not reflected in the *in vitro* assay.

10.1128/mBio.00365-18.5 FIG S5  Enhanced killing of C. albicans *in vitro* by BM cells treated with IFN-β. A total of 500,000 freshly isolated BM leukocytes from WT or IFIT2 KO mice were seeded in a well of a 96-well plate and infected with 100 C. albicans cells in the absence or presence of 1,000 U/ml murine IFN-β. Cultures were incubated overnight, and C. albicans colonies were quantified visually following staining with calcofluor white (Sigma) in 8% formaldehyde. Colonies were counted using UV light with Zeiss Microscope Observer D1, and the mean ± SEM from 16 wells was calculated. (Left) Image of one of the wells with C. albicans colony growth and BSA control cells from WT mice. (Right) Mean ± SEM of C. albicans colonies per well with cells from WT (light bars) or IFIT2 KO (dark bars) mice treated *in vitro* with BSA or IFN-β. Download FIG S5, PDF file, 0.1 MB. Copyright © 2018 Stawowczyk et al. 2018 Stawowczyk et al. This content is distributed under the terms of the Creative Commons Attribution 4.0 International license.

The first line of defense against C. albicans infection *in vivo* depends on the coordinate action of neutrophils, macrophages, dendritic cells, natural killer cells, innate-like lymphocytes, and epithelial cells (76). To determine whether a specific phagocyte lineage is responsible for improved survival of IFIT2 KO mice, we depleted mice of either neutrophils or monocytes and macrophages prior to infection. Neutrophil depletion resulted in increased fungal burden and pathogenesis in both WT and IFIT2 KO mice, and the IFIT2 KO mice were still better able to limit C. albicans replication in comparison with WT mice (Fig. 5A). In contrast, depletion of monocytes and macrophages with clodronate eliminated the differential ability of IFIT2 KO mice to reduce fungal burden (Fig. 5B). The kidney titer of C. albicans in clodronate-treated mice was significantly higher in IFIT2 KO mice than in WT mice (Fig. 5B). The results suggest the improved survival of IFIT2 KO mice is mediated by monocytes and macrophages.

IFNs are essential mediators of host defense, and yet they are also known to provoke pathological responses (13, 59, 60, 77). Since the elimination of just one ISG (IFIT2) led to increased survival of systemic C. albicans in mice, it suggested that type I IFNs could have a generalized detrimental effect. For this reason, we determined the influence of IFN-β administration during C. albicans infection. The most common approach to investigate the role of type I IFNs in murine models of disease has been to evaluate survival and pathology of mice that lack the gene encoding IFNAR1 (50, 78). Notably, experiments performed with systemic C. albicans infection of IFNAR1 KO mice have produced conflicting results. Studies reported IFNAR1 KO mice are either more susceptible to C. albicans infection (35, 36) or, alternatively, have increased survival of C. albicans infection (33, 34). Since type I IFNs are administered therapeutically to patients for the treatment of viral infections, cancer, and multiple sclerosis (11), we administered IFN-β to mice and determined their response to invasive C. albicans. IFN-β treatment was found to promote pathology and death from systemic C. albicans infection (Fig. 6). C. albicans titers in kidneys increased, and macrophage numbers in kidneys decreased with IFN-β treatment. Moreover, IFN-β treatment led to a dramatic increase in chemokine and cytokine production correlating with increased fungal burden in both WT and KO mice (Fig. 6 and 7; Tables S3 and S4). However, the fungal burden was consistently lower in IFIT2 KO mice, as it was without IFN-β treatment. The difference between WT and IFIT2 KO mice did not appear to be due to different IFN levels since both groups displayed similar levels of IFN-β and IFN-γ in the kidney lysates (Tables S2 and S4). While IFIT2 does not directly participate in regulation of IFN production, it is clearly involved in the response to IFN signaling. The moderate increase in chemokine and cytokine levels in infected IFIT2 KO mice without IFN-β administration correlated with protection; however, the dramatic boost of chemokine and cytokine production following IFN-β administration was deleterious. Proper regulation of IFN response is essential for development of a balanced immune response that is adequately strong to eliminate the pathogen but not severe enough to cause damage to the host. To our knowledge, this is the first study that directly evaluates the administration of type I IFN on the host response to C. albicans infection in a mouse model, and the results clearly caution the use of IFN therapy in patients at risk for infection.

An effective host defense against infection needs to strike the right balance between inflammatory responses that can be protective or harmful. Type I IFNs are critical mediators of innate immunity; however, our study indicates IFN-β treatment promotes fatal pathology during systemic C. albicans infection in mice and that IFIT2 is a mechanistic component that can contribute to this disease.

## MATERIALS AND METHODS

### Cell culture.

HeLa and 293T cells were obtained from ATCC. Mouse bone marrow cells and splenocytes were cultured in RPMI 1640 with 10% fetal bovine serum (FBS). Cells were stimulated with 1,000 U/ml human IFN-α (Roche) overnight.

### Plasmids and transfections.

cDNAs for human and murine genes were obtained from GE Dharmacon (formerly Open Biosystems) and subcloned into the HA-pCGN (Addgene) or pEF1-V5-HisB (Invitrogen) expression vectors. Polyethylenimine linear transfection reagent (Polysciences, Inc.) was used at 3 µg/µg plasmid DNA.

### Western blot, immunoprecipitation, and antibodies.

Cell lysates and Western blots were prepared as described previously (24). Immunoprecipitations were carried out with 600 to 1,000 µg protein with rabbit polyclonal anti-mouse ISG54 (24), rabbit polyclonal anti-HA (Santa Cruz), mouse monoclonal anti-HA (Sigma), mouse monoclonal anti-V5 (Santa Cruz), mouse monoclonal anti-actin antibody (Sigma), and control normal rabbit IgG (Santa Cruz Biotechnology). Commercial secondary antibodies included horseradish peroxidase (HRP)-conjugated anti-mouse and anti-rabbit antibodies (GE Healthcare) and anti-mouse IRDye800-conjugated antibody (Rockland).

### Mice and C. albicans infection.

Mice were bred and housed at the Stony Brook University Division of Laboratory Animal Research facility. Protocols were approved by the Institutional Animal Care and Use Committee. Mice with a null allele in *ifit2*/*isg54* were generated by the NIH Knockout Mouse Project (KOMP) (Ifit2_AA5) in C57BL/6N mice from Charles River Laboratories, Inc. Mice used for experiments were 8 to 10 weeks old and were inoculated into the lateral tail vein with 2.5 × 10^5^ cells of the C. albicans WT strain (SC5314) (79). IFN-β treatment was performed by intraperitoneal injection of 1 × 10^5^ U murine IFN-β in 0.1 µg BSA/ml phosphate-buffered saline (PBS; 0.2 ml), and controls received 0.1 µg BSA/ml PBS (0.2 ml) 16 to 18 h prior to infection. Recombinant IFN-β with a specific antiviral activity of 2 × 10^8^ U/mg was obtained from Biogen, Inc. (Cambridge, MA).

### Immunohistochemistry.

Kidneys from WT or IFIT2 KO mice were isolated at 72 hpi, fixed overnight in 4% paraformaldehyde, and processed by Histowiz, Inc. (Brooklyn, NY). Digital images were visualized using ImageScope software (Leica).

### Isolation of bone marrow cells, splenocytes, and kidney cells.

Bone marrow (BM) cells were isolated by flushing tibias and femurs, and spleens were disrupted mechanically between frosted microscope slides before red blood cell lysis. Kidneys were disrupted mechanically, and resuspended fragments were treated as described with DNase and Liberase enzyme (Roche) (80).

### Neutrophil, monocyte, and macrophage depletion.

To deplete neutrophils, 50 µg of anti-Ly6G antibody clone 1A8 (BioXCell) was injected intravenously in tail veins 24 h before infection. The efficiency of depletion was evaluated at the time of sacrifice in peripheral blood. Leukocytes were stained with anti-Gr1 antibody (BioLegend) and analyzed with flow cytometry. Depletion of monocytes and macrophages was carried out by intravenous tail vein injection with 200 µl clodronate liposomes 24 h before infection. Clodronate liposomes were obtained from http://clodronateliposomes.org and used according to the manufacturer’s instructions.

### Cytokine and chemokine assays.

Analysis of chemokines and cytokines was carried out using LEGENDplex multianalyte flow assay kits (BioLegend). Chemokines were analyzed with mouse proinflammatory chemokine panel, and cytokines were analyzed with a BioLegend mouse inflammation panel (BioLegend) with a FACSCalibur cytometer (BD Biosciences).

### Blood cell type analysis of kidneys.

Kidney cells were harvested and incubated with the following fluorophore-conjugated antibodies (BioLegend): Pacific Blue–anti-CD45, brilliant violet 650–anti-Ly6G, APC/Cy7–anti-F4/80, and APC–anti-Ly6C. Cells were blocked with anti-mouse CD16/32 antibody at 1:50 dilution for 30 min at 4°C, resuspended, and added to antibody pellets followed by fixation with 2% paraformaldehyde. Analysis was performed using an LSRII flow cytometer (BD Biosciences).

### Phagocytosis assays.

Phagocytosis of live C. albicans cells was measured with GFP-expressing C. albicans (*Ca*GFP) cells (47). Freshly isolated BM cells were stained with anti-CD45 antibody (BioLegend), were untreated, or were treated with 1,000 U/ml murine IFN-β, and 3 × 10^5^ BM cells were incubated with 1.5 × 10^6^ of GFP-expressing C. albicans cells. After 20 min, phagocytosis was stopped with paraformaldehyde. Samples were analyzed with a FACSCalibur flow cytometer by calculating the percentage of CD45-positive cells that were also GFP positive. A modification of the published assay to measure phagocytosis of heat-killed C. albicans was used (81). BM cells were left untreated or stimulated with murine IFN-β (1,000 U/ml). C. albicans cells were heat inactivated at 95°C for 30 min, stained with SYBR-Safe DNA stain (Thermo Fisher), and washed, and 1 × 10^6^ yeast cells were added to 1 × 10^6^ prepared BM cells. After 30 min, propidium iodide (Invitrogen) was added to quench extracellular green fluorescence. Green fluorescence in the BM cells was measured with a FACSCalibur flow cytometer (BD Biosciences).

### Measurement of reactive oxygen species.

Freshly isolated BM cells were incubated in phenol red-free complete RPMI and were left untreated or were stimulated with murine IFN-β (1,000 U/ml) followed by staining with 20 µM DCFDA (2′,7′-dichlorofluorescin diacetate; Abcam, Inc.). A total of 1 × 10^6^ BM cells were incubated with 1 × 10^4^ C. albicans cells. Green fluorescence was measured with a FACSCalibur flow cytometer by calculating geometric mean fluorescence per cell.
