# Supplementary material for: Pathogenic Effects of IFIT2 and Interferon-β during Fatal Systemic Candida albicans Infection
Source: mBio. 2018 Apr 17;9(2):e00365-18. doi: 10.1128/mBio.00365-18 (PMC5904408; doi:10.1128/mBio.00365-18)
Supplement: FIG S1 [file mbo002183841sf1.pdf]

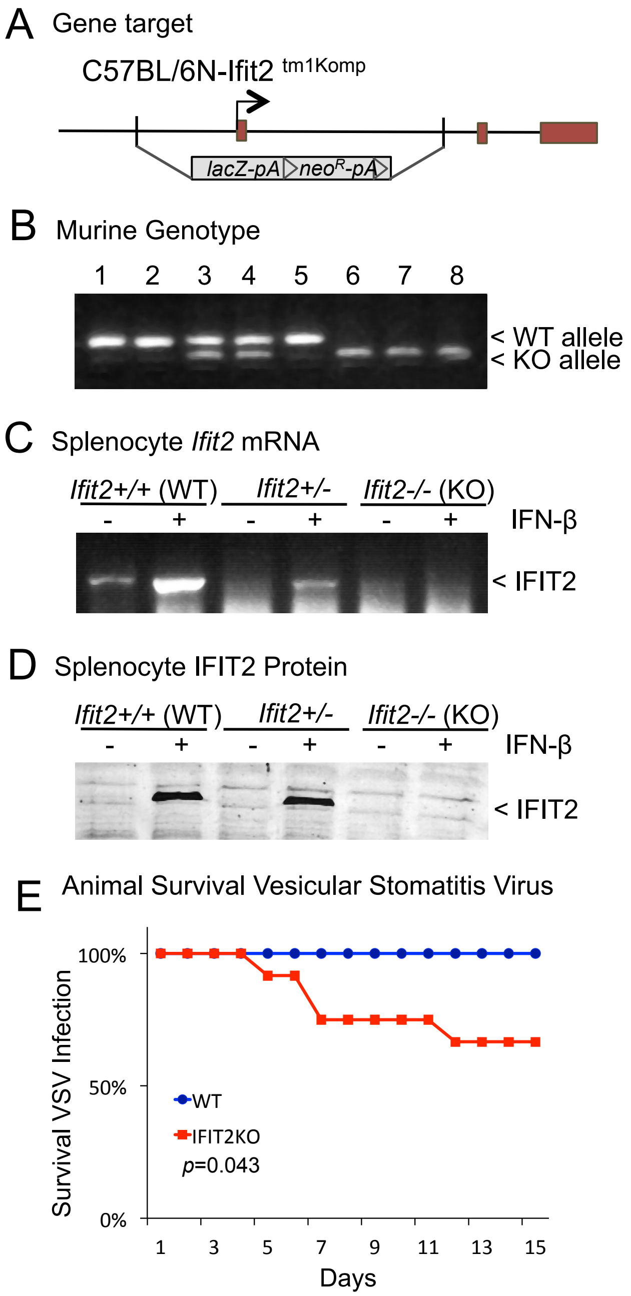

Figure S1. Murine IFIT2 gene knockout. Mice with a null allele in *ifit2/isg54* were generated by the NIH Knock-Out Mouse Project (KOMP)(Ifit2_AA5). **A)** Diagram of *ifit2* deletion strategy and replacement of promoter and first exon with lacZ-neo cassette. KO generated in C57Bl/6N mice from Charles River Laboratories. Fragment of chromosome 19 encompassing sequence 34,616,691-34,642,416 was deleted using insert consisting of lacZ gene and loxP-flanked neo-cassette. Promoter, transcription start site and first exon were deleted. Arrow indicates transcriptional start site. **B)** Genotype results of eight mice either homozygous for wild-type (WT) *ifit2* alleles, or heterozygous or homozygous for knockout (KO) alleles. **C)** IFIT2 mRNA expression measured in splenocytes by RT-PCR. **D)** IFIT2 protein expression evaluated by Western blot of murine splenocyte lysates prepared from homozygous WT *ifit2* (+/+), heterozygous *ifit2* (+/-), or homozygous KO *ifit2* (-/-) mice (anti-mIFIT2 serum). Cells were untreated or treated with 1,000 U// mL murine IFN-b for two hours. IFIT2 protein levels were similar in WT or heterozygous cells. **E)** Kaplan-Meier survival curves of WT mice or IFIT2 KO mice following intranasal infection with Vesicular Stomatitis virus (VSV). Animals were sedated with isofluorane (Isothesia, Butler Schein) and infected intranasally with Vesicular Stomatis Virus (VSV) in 10 uL volume of complete DMEM. VSV was obtained from ATCC, propagated in Vero cells and the stock titer was determined by plaque assay. Survival of animals was monitored daily. Two independent experiments were performed and a total number of 12 WT and 12 IFIT2 KO mice were used. Graph shows results from one representative experiment with six WT and IFIT2 KO mice and 5×10^6 pfu VSV. *p* value determined by the log-rank test.
