# Supplementary material for: Pathogenic Effects of IFIT2 and Interferon-β during Fatal Systemic Candida albicans Infection
Source: mBio. 2018 Apr 17;9(2):e00365-18. doi: 10.1128/mBio.00365-18 (PMC5904408; doi:10.1128/mBio.00365-18)
Supplement: FIG S2 [file mbo002183841sf2.pdf]

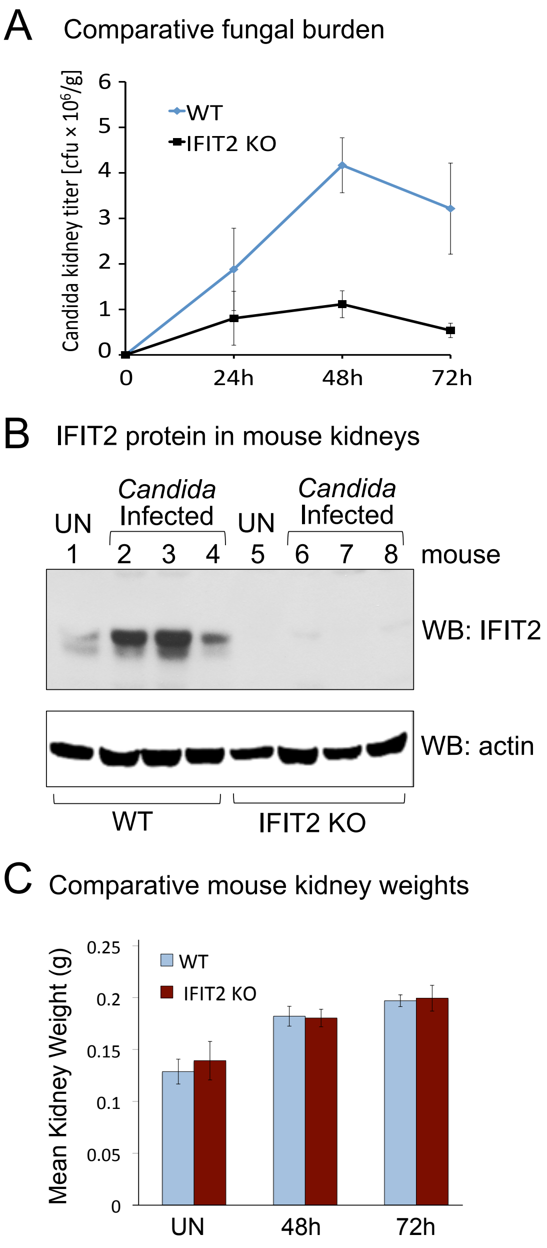

Figure S2. Response of WT and IFIT2 KO mice to *C. albicans* infection. A) Kidney fungal burden in WT and IFIT2 KO mice at 24, 48, and 72 h.p.i. The mean of data is shown at 24h.p.i for 14 WT and 14 IFIT2KO mice, at 48 h.p.i. for 24 WT and 22 IFIT2 KO mice, and at 72 h.p.i. for 22 WT and 22 IFIT2 KO mice, from 4 independent experiments (SEM). **B)** Western blot of IFIT2 protein expression in kidney lysates from uninfected (UN) or *C. albicans*-infected WT or IFIT2 KO mice. Kidneys were harvested 72 hours post infection and polyclonal anti-mIFIT2 was used to detect protein. Numbers represent individual mice. **C)** Weights of kidneys isolated from WT (light bars) or IFIT2 KO (dark bars) mice uninfected or infected with *C. albicans* for 48 or 72 h.
