# Supplementary material for: Pathogenic Effects of IFIT2 and Interferon-β during Fatal Systemic Candida albicans Infection
Source: mBio. 2018 Apr 17;9(2):e00365-18. doi: 10.1128/mBio.00365-18 (PMC5904408; doi:10.1128/mBio.00365-18)
Supplement: TABLE S1 [file mbo002183841st1.pdf]

Table S1: Chemokine Profile of Kidneys from Uninfected and *C. albicans*-Infected C57Bl/6 WT mice and IFIT2 knockout mice, 48 or 72 hours post infection. Mean from 19-22 infected mice (5 independent experiments with 4-5 mice) presented in pg/ml as described in Materials and Methods.

	**WT**			**IFIT2 KO**		
Chemokine	UN	48hr pi	72hr pi	UN	48hr pi	72hr pi
CCL2 /MCP1	6.8	27.4	23.1	6.2	**47.0***	**47.1***
CCL3 /MIP-1α	0.9	12.9	27.5	0.9	**86.8****	82.7
CCL4 /MIP-1β	0.9	15.6	20.5	0.9	**49.3***	63.0
CCL5 /RANTES	26.9	29.5	37.2	24.9	39.2	28.9
CCL11 /Eotaxin	2.1	6.7	10.4	1.6	**17.5***	19.1
CCL17 /TARC	3.5	2.4	2.7	2.4	2.7	2.1
CCL20 /MIP-3α	21.3	44.6	52.4	17.7	**103.1****	**92.5***
CCL22 /MDC	60.0	33.6	45.9	63.0	46.3	33.6
CXCL1 /KC	13.9	68.1	131.7	12.1	**125.2***	**198.9***
CXCL5 /LIX	9.3	14.3	30.8	6.5	**48.2****	**59.5****
CXCL9 /MIG	17.7	36.7	23.7**	11.7	46.2	8.2
CXCL10 /IP-10	0.9	1.2	3.1*	0.9	3.5*	1.8
CXCL13 /BLC	44.8	363.7	206.0	42.9	**830.1***	**524.3***

Significant difference KO vs. WT, * $p < 0.05$ ** $p < 0.01$
