# Supplementary material for: Pathogenic Effects of IFIT2 and Interferon-β during Fatal Systemic Candida albicans Infection
Source: mBio. 2018 Apr 17;9(2):e00365-18. doi: 10.1128/mBio.00365-18 (PMC5904408; doi:10.1128/mBio.00365-18)
Supplement: TABLE S2 [file mbo002183841st2.pdf]

Table S2: Cytokine Profile of Kidneys from Uninfected and *C. albicans*-Infected C57Bl/6 WT mice and IFIT2 knockout mice, 48 or 72 hours post infection. Mean from 12-22 infected mice (3-4 independent experiments with 3-6 mice) presented in pg/ml as described in Materials and Methods.

	**WT**			**IFIT2 KO**		
Cytokine	UN	48hr pi	72hr pi	UN	48hr pi	72hr pi
IL-1α	1.7	250.9	379.5	1.0	166.1	727.3
IL-1β	2.7	50.6	60.0	2.5	48.9	67.0
IL-6	40.5	61.7	282.7	37.1	74.6	703.8
IL-10	2.9	32.8	36.2	1.2	33.3	55.2
IL-12p70	5.7	7.3	5.4	6.2	6.9	4.7
IL-17α	18.8	6.7	5.7	16.1	5.6	5.5
IL-23	7.3	12.8	14.4	7.4	12.6	14.6
IL-27	2.8	74.9	55.6	1.5	79.4	50.9
IFN-β	7.5	7.2	6.6	6.3	7.3	6.3
IFN-γ	3.8	8.5	4.2	3.9	4.8	4.3
GM-CSF	18.3	33.5	26.4	14.8	33.2	25.0
TNF-α	1.4	24.4	25.9	1.4	23.7	33.9
