# Supplementary material for: Pathogenic Effects of IFIT2 and Interferon-β during Fatal Systemic Candida albicans Infection
Source: mBio. 2018 Apr 17;9(2):e00365-18. doi: 10.1128/mBio.00365-18 (PMC5904408; doi:10.1128/mBio.00365-18)
Supplement: FIG S3 [file mbo002183841sf3.pdf]

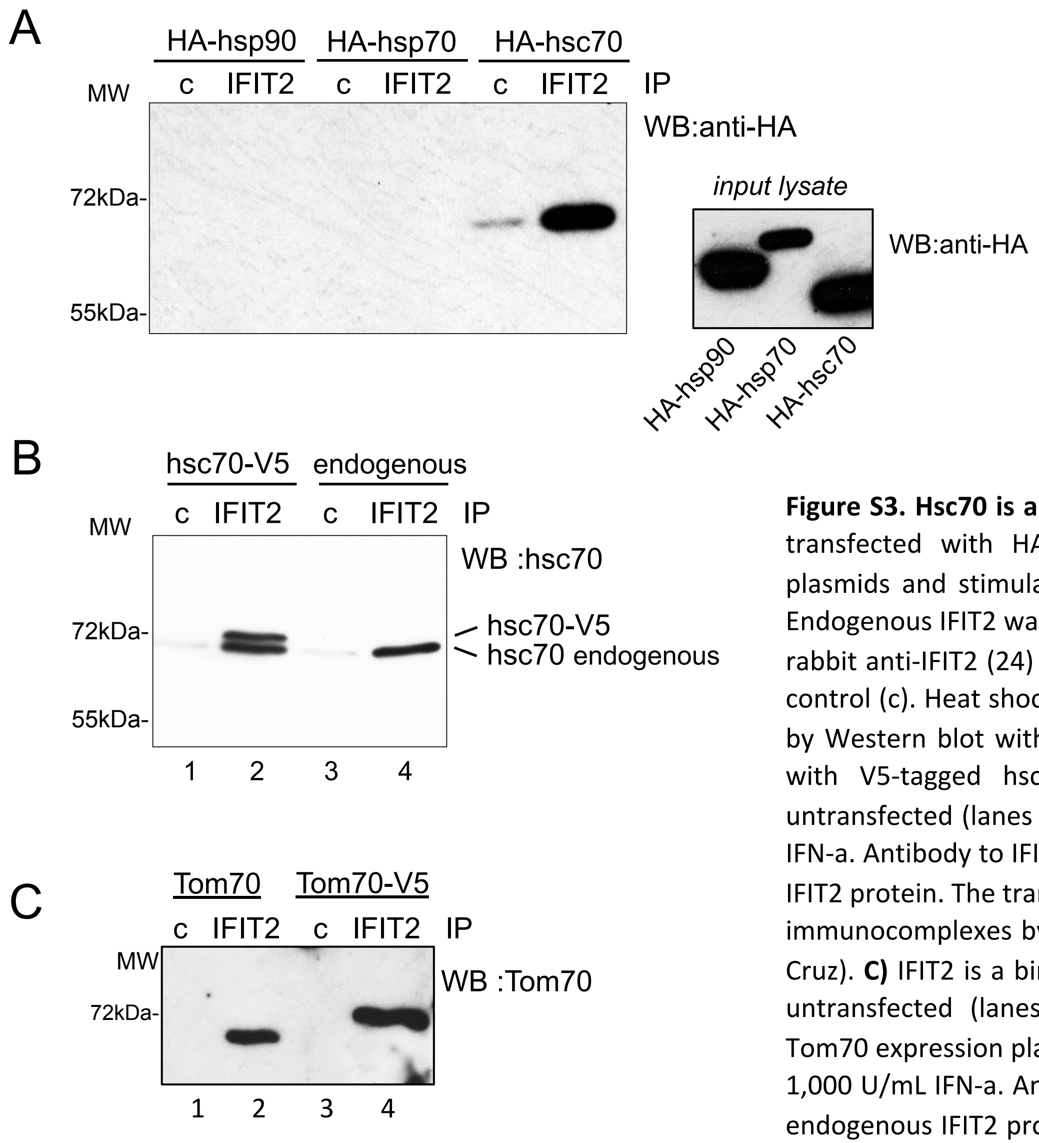

Figure S3. Hsc70 is a binding partner with IFIT2. A) HeLa cells were transfected with HA-tagged hsp90, hsp70, or hsc70 expression plasmids and stimulated overnight with 1,000 U/mL human IFN-a. Endogenous IFIT2 was immunoprecipitated (IP) from cell lysates with rabbit anti-IFIT2 (24) and non-specific rabbit antibody was used as a control (c). Heat shock proteins were detected in immunocomplexes by Western blot with anti-HA antibodies. **B)** Cells were transfected with V5-tagged hsc70 expression plasmid (lanes 1,2), or left untransfected (lanes 3,4) and stimulated overnight with 1,000 U/mL IFN-a. Antibody to IFIT2 was used to immunoprecipitate endogenous IFIT2 protein. The transfected or endogenous hsc70 were detected in immunocomplexes by Western blot with anti-hsc70 antibody (Santa Cruz). **C)** IFIT2 is a binding partner with Tom70. HeLa cells were left untransfected (lanes 1, 2), or were transfected with V5-tagged Tom70 expression plasmid (lanes 3, 4) and stimulated overnight with 1,000 U/mL IFN-a. Antibody to IFIT2 was used to immunoprecipitate endogenous IFIT2 protein. Both transfected and endogenous Tom70 were detected in immunocomplexes by Western blot with anti-Tom70 antibody (Santa Cruz).
