# Supplementary material for: Pathogenic Effects of IFIT2 and Interferon-β during Fatal Systemic Candida albicans Infection
Source: mBio. 2018 Apr 17;9(2):e00365-18. doi: 10.1128/mBio.00365-18 (PMC5904408; doi:10.1128/mBio.00365-18)
Supplement: FIG S4 [file mbo002183841sf4.pdf]

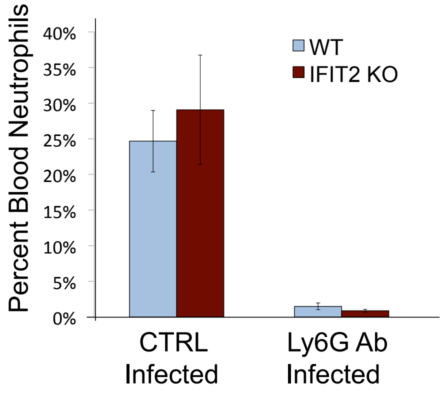


**Figure S4. Evaluation of effectiveness of neutrophil depletion by anti-Ly6G administration.**

Anti- Ly6G antibodies were administered by intravenous injection 24 hours prior to infection of WT (light bars) or IFIT2 KO (dark bars) mice with *C. albicans*. Mice were sacrificed 48 h.p.i. and the percent of neutrophils in the blood was measured by flow cytometry with anti-Gr1 antibodies (Biolegend). SEM
