# Supplementary material for: Pathogenic Effects of IFIT2 and Interferon-β during Fatal Systemic Candida albicans Infection
Source: mBio. 2018 Apr 17;9(2):e00365-18. doi: 10.1128/mBio.00365-18 (PMC5904408; doi:10.1128/mBio.00365-18)
Supplement: TABLE S3 [file mbo002183841st3.pdf]

Table S3: Chemokine Profile of Kidneys from C57Bl/6 WT mice and IFIT2 KO mice treated with BSA or IFN-β and subsequently infected with *Candida albicans* for 72 hours. Mean from 5 infected mice presented in pg/ml as described in Materials and Methods.

	**WT**		**IFIT2 KO**	
Chemokine	+BSA	+IFN	+BSA	+IFN
CCL2 /MCP1	38.7	111.6§	60.5	100.4
CCL3 /MIP-1α	59.8	364.0§	71.6	**619.4***§
CCL4 /MIP-1β	61.6	313.3§	83.7	319.3§
CCL5 /RANTES	31.7	41.4	23.5	44.8§
CCL11 /Eotaxin	19.7	51.6§	25.5	**79.0***§
CCL17 /TARC	2.3	2.0	1.8	3.0§
CCL20 /MIP-3α	73.5	181.4§	120.7	217.4§
CCL22 /MDC	33.5*	39.0	23.0	55.8§
CXCL1 /KC	160.2	285.8§	214.9	**404.6***§
CXCL5 /LIX	45.4	74.8§	75.3	**133.1***§
CXCL9 /MIG	18.0*	12.3	4.5	66.2§
CXCL10 /IP-10	3.6	2.7	2.9	**4.5***§
CXCL13 /BLC	325.4	1192.0§	559.3*	**3331.5***§

* Significant difference KO vs. WT, $p<0.05$
§ Significant increase with IFN treatment, $p<0.05$
