# Supplementary material for: Pathogenic Effects of IFIT2 and Interferon-β during Fatal Systemic Candida albicans Infection
Source: mBio. 2018 Apr 17;9(2):e00365-18. doi: 10.1128/mBio.00365-18 (PMC5904408; doi:10.1128/mBio.00365-18)
Supplement: TABLE S4 [file mbo002183841st4.pdf]

Table S4: Cytokine Profile of Kidneys from BSA or Interferon-β (IFN) treated C57Bl/6 WT mice or IFIT2 KO mice infected with *Candida albicans* for 72 hours. Mean from 5 infected mice presented in pg/ml as described in Materials and Methods.

	**WT**		**IFIT2 KO**	
Cytokine	BSA	IFN	BSA	IFN
IL-1α	642.1	2396.6§	787.2	2326.3§
IL-1β	55.0	162.4§	77.7	157.9§
IL-6	352.7	2602.6§	369.0	**5374.9***§
IL-10	29.3	126.6§	24.0	157.6§
IL-12p70	3.1	7.2	2.7	7.4§
IL-17α	4.0	7.3§	3.7	9.7§
IL-23	9.6	17.2§	12.9	18.6
IL-27	14.7	22.0	11.9	29.1§
IFN-β	3.3	6.2§	3.0	6.2§
IFN-γ	2.7	4.5§	2.7	5.6§
GM-CSF	5.1	11.3	5.4	14.1§
TNF-α	22.4	61.3§	21.6	74.5§

* Significant difference KO vs. WT, $p<0.05$
§ Significant increase with IFN treatment, $p<0.05$
