# Supplementary material for: Pathogenic Effects of IFIT2 and Interferon-β during Fatal Systemic Candida albicans Infection
Source: mBio. 2018 Apr 17;9(2):e00365-18. doi: 10.1128/mBio.00365-18 (PMC5904408; doi:10.1128/mBio.00365-18)
Supplement: FIG S5 [file mbo002183841sf5.pdf]

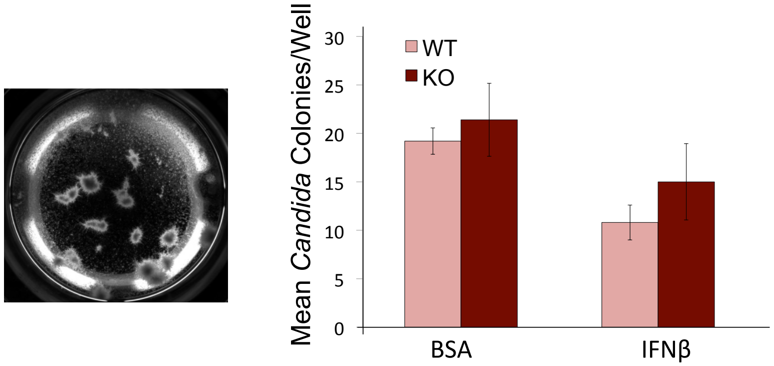

Figure S5. Enhanced killing of *C. albicans in vitro* by BM cells treated with IFN-β. 500,000 freshly isolated BM leukocytes from WT or IFIT2 KO mice were seeded in a well of a 96-well plate and infected with 100 *C. albicans* cells in the absence or presence of 1000 U/mL murine IFN-β. Cultures were incubated overnight and *C. albicans* colonies were quantified visually following staining with Calcofluor White (Sigma) in 8% formaldehyde. Colonies were counted using UV light with Zeiss Microscope Observer D1 and the mean from 16 wells was calculated (SEM). Left) Image of one of the wells with *C. albicans* colony growth and BSA control cells from WT mice. Right) Mean of *C. albicans* colonies per well with cells from WT (light bars) or IFIT2 KO (dark bars) mice treated in vitro with BSA or IFN-β.
